# Pathogenesis Based Diagnosis and Treatment of Endometriosis

**DOI:** 10.3389/fendo.2021.745548

**Published:** 2021-11-25

**Authors:** Philippe R. Koninckx, Rodrigo Fernandes, Anastasia Ussia, Larissa Schindler, Arnaud Wattiez, Shaima Al-Suwaidi, Bedayah Amro, Basma Al-Maamari, Zeinab Hakim, Muna Tahlak

**Affiliations:** ^1^ Latifa Hospital, Dubai, United Arab Emirates; ^2^ Prof Emeritus Obstet Gynecol (OBGYN), Catholic University Leuven (KU), Leuven, Belgium; ^3^ University of Oxford-Hon Consultant, Oxford, United Kingdom; ^4^ University Cattolica, Roma, Italy; ^5^ Moscow State University, Moscow, Russia; ^6^ Gruppo Italo Belga, Villa Del Rosario, Rome, Italy; ^7^ Instituto do Cancer do Estado de São Paulo, University of São Paulo, São Paulo, Brazil; ^8^ Dubai Fertility Centre of the Dubai Health Authority, Dubai, United Arab Emirates; ^9^ Prof Department of Obstetrics and Gynaecology, University of Strasbourg, Strasbourg, France

**Keywords:** endometriosis, endometriosis natural history, endometriosis diagnosis, endometriosis prevention, endometriosis treatment, endometriosis surgery

## Abstract

Understanding the pathophysiology of endometriosis is changing our diagnosis and treatment. Endometriosis lesions are clones of specific cells, with variable characteristics as aromatase activity and progesterone resistance. Therefore the GE theory postulates GE incidents to start endometriosis, which thus is different from implanted endometrium. The subsequent growth in the specific environment of the peritoneal cavity is associated with angiogenesis, inflammation, immunologic changes and bleeding in the lesions causing fibrosis. Fibrosis will stop the growth and lesions look burnt out. The pain caused by endometriosis lesions is variable: some lesions are not painful while other lesions cause neuroinflammation at distance up to 28 mm. Diagnosis of endometriosis is made by laparoscopy, following an experience guided clinical decision, based on history, symptoms, clinical exam and imaging. Biochemical markers are not useful. For deep endometriosis, imaging is important before surgery, notwithstanding rather poor predictive values when confidence limits, the prevalence of the disease and the absence of stratification of lesions by size, localization and depth of infiltration, are considered. Surgery of endometriosis is based on recognition and excision. Since the surrounding fibrosis belongs to the body with limited infiltration by endometriosis, a rim of fibrosis can be left without safety margins. For deep endometriosis, this results in a conservative excision eventually with discoid excision or short bowel resections. For cystic ovarian endometriosis superficial destruction, if complete, should be sufficient. Understanding pathophysiology is important for the discussion of early intervention during adolescence. Considering neuroinflammation at distance, the indication to explore large somatic nerves should be reconsidered. Also, medical therapy of endometriosis has to be reconsidered since the variability of lesions results in a variable response, some lesions not requiring estrogens for growth and some being progesterone resistant. If the onset of endometriosis is driven by oxidative stress from retrograde menstruation and the peritoneal microbiome, medical therapy could prevent new lesions and becomes indicated after surgery.

## Highlights

Endometriosis initiates after genetic or epigenetic (GE) changes in an endometrial, stem or bone marrow cell. The GE defects inherited at birth explain the endometriosis predisposition, the associated infertility and some immunologic changes. The probability of initiating endometriosis is highest after puberty, because of estrogens and initiating menstruation and sexual activity, and decreases progressively thereafter. The growth of lesions is clonal and variable, but self-limiting, probably because of fibrosis. The pathophysiology of endometriosis thus is incompatible with the implantation of normal endometrial cells. Endometriosis thus should no longer be considered a progressive and recurrent disease.

These concepts could permit the prevention of endometriosis by reducing the oxidative stress from retrograde menstruation and the upper genital tract and peritoneal microbiome. This might be achieved by oral contraceptives taken continuously and by manipulating the intestinal microbiome through food intake and exercise, and eventually by anti-oxidants.

That endometriosis lesions are clonal and molecularly different, can explain that only some lesions cause pain at distances up to 28 mm. Also, if lesions are heterogeneous, the effect of medical therapy cannot be predicted by traditional statistics. Heterogeneity also explains that lesions with strong aromatase activity do not need circulating estrogens for growth and that in lesions with progesterone resistance, progesterone treatment is not effective.

Diagnosis of endometriosis before and during surgery is based on clinical experience which integrates age, symptoms, clinical exam, imaging and recognition during surgery.

The treatment of choice is surgery. However, surgery should be less radical if the surrounding fibrosis is not endometriosis, and if small microscopical endometriotic nests and eventually the periphery of deep nodules are cells with reversible metaplastic changes, which return to normal after excision.

## Introduction

Endometriosis is a frequent disease, affecting over 10% of women. It is a major cause of infertility and pelvic pain. However, definitions and prevalences have changed over time. Endometriosis was described in 1860 as endometrium like cells in the myometrium (1), in 1897 as severe lesions in the rectovaginal septum (2) then called adenomyoma, as ovarian chocolate cysts in 1921 (3) and somewhat later as typical black puckered typical lesions. After 1940 the number of case reports increased exponentially describing many endometriosis lesions found accidentally during surgery, such as endometriosis of the sciatic nerve (4). In 1950 endometriosis had become the most frequent cause for surgery and hysterectomy in women (5), even after menopause (6). With the introduction of laparoscopy, the high prevalence in women with pain and infertility was realized. In 1986, non-coloured lesions were recognised as subtle endometriosis (7) and in 1990 deep endometriosis was described (8). The estimated incidences of endometriosis thus changed over time. Moreover, epidemiology and differences between races and countries (9) remain poorly known (10, 11), since the diagnosis of superficial lesions requires a laparoscopy and since recognition, especially of subtle endometriosis, is variable (12). Many endometriosis lesions probably are not recognised, since, in women with endometriosis, 30% of normal-looking appendices harbour endometriosis (13) and since in women with a deep endometriosis lesion of the bowel harbour microscopical foci of endometriosis at distance in the bowel (14, 15) and the lymph nodes (16). Endometriosis and adenomyosis are believed to be associated, although the supporting data are circumstantial (17). Adenomyosis is variable from a thickened or irregular junctional zone, to diffuse adenomyosis in the myometrium to focal nodular adenomyosis (18). Regional and ethnic differences have been poorly investigated. Larger nodular adenomyosis lesions, as an example, are rare in northern Europe, but more frequent in Greece (18) and the south of Italy.

The literature on endometriosis and adenomyosis comprises over 30.000 peer-reviewed articles, numerous books, websites and lay articles. Endometriosis meetings are frequent and endometriosis patient associations exist in almost every country. This signals that information is not clear, that many women are not happy with the diagnosis and treatment, and that symptoms, diagnosis and treatment remain discussed. Moreover, when for a multi-faceted disease like endometriosis the evidence is not clear, alternative hypotheses and therapies emerge such as acupuncture (19), herbal medicine (20), dietary changes (21), physical medicine and anti-inflammatory and anti-oxidant (22) therapy.

The information on endometriosis varies with the endometriosis specialist, and not all of them have a full understanding of various aspects as genetics, statistics, endocrinology, imaging, medical therapy, surgery and alternative therapies. The endometriosis specialists vary from the infertility specialist, the pain specialist, and the endometriosis surgeon, each of them with specific expertise, and a variable diagnosis and therapy (23). Books on endometriosis are mostly written by several authors, with their specific interests and biases, resulting in differences in opinion and emphasis, which may sound like contradictions.

Mistakes of diagnosis and surgery cannot be investigated for ethical reasons and therefore information is not available. Although complications of the surgery are well documented, we do not have information on surgery or medical treatment that should not have been performed or that was performed for the wrong indications.

## The Pathophysiology of Endometriosis

### Epigenetics

All cells of the human body develop from one fertilized oocyte and carry the same genetic DNA information. However, cell division and DNA duplication is a complex process with the risk high of mistakes. DNA mistakes are either repaired or if too serious, the cell becomes apoptotic. However, small mistakes can remain and are transmitted to the next generation of cells as is known in oncology and benign tumours as myoma’s or polyps.

The long strands of DNA are coiled around nucleosomes and functionally organised by surrounding histone proteins and selective methylation. Thus parts of DNA become less or more accessible and functional, creating a differentiated cell (24, 25). The epigenetic memory organises the new cell as the parent cell. Epigenetics, therefore, is defined as non-DNA transmissible information. The mechanism is still poorly understood, but could be compared to folding origami: the original paper is intact but will be folded easily in a similar way as previously, although folding differently remains possible.

The epigenetic organisation and activity of our DNA are transmissible. Some changes are reversible, but similar to a long rope becoming easily twisted, complex epigenetic differentiation becomes irreversible, difficult to untangle although not broken (26).

### The Endometrium

The endometrium is the fastest-growing differentiated tissue of the human body, with thus a higher risk of mitotic mistakes. Fortunately, most are eliminated during menstruation. The menstrual cycle is a strictly timed cascade of events with follicular growth and increasing estrogen production, ovulation and progesterone secretion by the corpus luteum. Estrogens stimulate the growth of the endometrium and induce progesterone receptors. Progesterone stops the growth and induces glandular changes and decidualisation, in preparation for implantation. When the concentrations of both estrogens and progestagens suddenly decrease the uterine endometrium is shedded and bleeding occurs. However, the endocrinology of the endometrium is much more complex. Estrogens are an equilibrium of estradiol and estrone. It is not that clear what the role is of androgens, 5a-reduced progestins, prostaglandins and cytokines. The endocrinology of the basal endometrium is poorly understood. It seems less responsive to estrogens while having some progesterone resistance.

The endometrium rests upon and has a specific relationship with the junctional zone as illustrated by the physiologic changes of the radial arteries during pregnancy. In the absence of these changes, women risk developing pre-eclampsia (27). Endometrial stromal cells have increased invasiveness, which seems to be kept balanced by the junctional zone (28).

### The Peritoneal Cavity

The peritoneal cavity does not belong to the core body and has to be considered a cavity outside the body, without vascularization like the mouth (29). In women, the peritoneal cavity has a direct connection to the outside world through the upper genital tract, uterus and cervix.

The peritoneal cavity is a specific micro-environment with a specific microbiome, influenced by the microbiome of the upper genital tract and the uterus during retrograde menstruation (30) and by the microbiome of the intestinal tract. In women, the peritoneal fluid is mainly an ovarian exudate, with changes during the menstrual cycle, and volumes up to 400 ml periovulatory. Being an ovarian exudate (31), the concentrations of proteins and steroid-binding molecules is 30% less than in plasma while the concentrations and time courses of estrogens and progestogens are very different. In the follicular phase concentrations of estrogens are higher than in plasma and concentrations of progesterone are much higher, comparable to luteal phase plasma concentrations. During ovulation, the follicular content is released into the peritoneal cavity and the ruptured follicle/early corpus luteum drains high amounts of steroid hormones with concentrations in the peritoneal cavity 100 to 1000 times higher than in plasma (32–34). Also, the immunology of the peritoneal cavity is specific and different from plasma with different concentrations of macrophages and cytokines (35). This is also important for peritoneal repair and adhesion formation after surgery (36). Retrograde menstruation (37), results in an overload of iron causing, together with the microbiome, oxidative stress (38, 39).

The endometriosis cells implanted superficially on the peritoneum grow and develop in the peritoneal fluid which is a specific micro-environment of estrogens, progestins, cytokines, growth factors, growth hormones, angiogenic factors and many more (32, 35). It thus is logical that endometriosis lesions look histologically different and not in phase with the endometrium. It remains strange that subtle lesions can be proliferative with active growth, notwithstanding an environment with relatively high progesterone concentrations, suggesting progesterone resistance.

Similarly, hormonal differences should be considered for cystic ovarian endometriosis, developing in the ovary. The intra-ovarian hormone concentrations, although poorly investigated, are estimated to be 100 to more than 1000 times higher than in plasma.

The superficial layers of deep endometriosis are influenced mainly by peritoneal fluid while deeper layers must be more influenced by plasma hormone concentrations. It thus is not surprising that deep layers of deep endometriosis are more active and in phase with the endometrium (8). *This* observation, together with a biphasic depth of distribution was one of the arguments to define deep endometriosis as endometriosis deeper than 5 mm (40).

### Pathophysiology: The Onset of Endometriosis

The hypotheses varied over time with the observations and their understanding. The first hypothesis, in the nineteenth century, was that endometriosis lesions were metaplastic changes, which is a histological description indicating the transformation of a differentiated cell into another differentiated cell (41). Later, metaplasia of embryological remnants was described for specific types of endometriosis (42, 43). Today metaplasia is understood as epigenetic changes transforming a differentiated cell, or a stem cell or a bone marrow cell, into another differentiated cell. Varying with the type of epigenetic changes, metaplasia thus can be reversible or irreversible.

In 1925 Sampson described the hypothesis of retrograde menstruation and implantation (44, 45). This hypothesis became popular since the endometrial cells are viable with implantation potential. However, when it was realised that retrograde menstruation occurred in almost all women (37), it became difficult to explain that not all women developed endometriosis. The recognition of subtle endometriosis lesions considered the early stages after implantation, confused till today. It stimulated the search for smaller or microscopical endometriosis occurring in normal looking peritoneum, in the bowel at distance from a nodule and in lymph nodes. They were considered a physiological phenomenon occurring intermittently in all women (46), disappearing and reappearing in other places (47, 48). Subtle lesions can cause pain (49). Although progression from subtle or other forms of endometriosis to more severe forms has never been demonstrated or observed, the concept of 1 progressive disease remains widely accepted and is reflected in the American fertility society classification of endometriosis (50).

**Figure 1:**
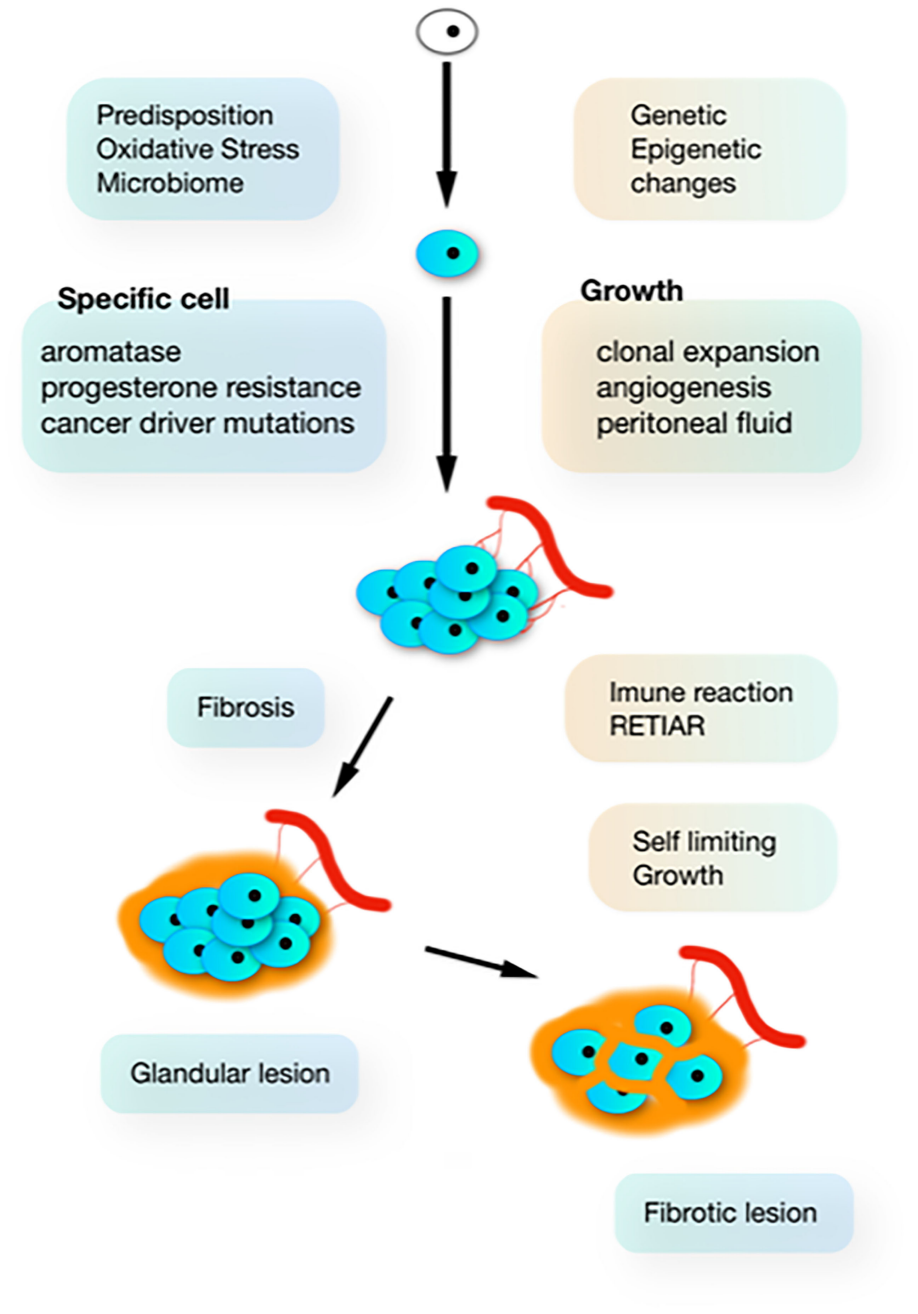
The natural history of endometriosis. In susceptible women, additional G-E incidents (driven by oxidative stress from retrograde menstruation and infection) induce clonal and heterogenous hetrogeneous (aromatase and progesterone resistance) lesions, which develop in the peritoneal cavity which is a specific microenvironment. Inhibition of growth by progressive fibrosis (because of immunology and bleeding in the lesions) results in variable severity of lesions.

The Sampson implantation theory is incompatible with the biological variability (51) and the clonal aspect (52–54) of endometriosis lesions. Each typical, cystic ovarian or deep lesion is a clonal tumour starting from one cell, and if a woman has 10 different lesions, these are 10 different clones (**Figure 1**). This clonal aspect explains why lesions are heterogeneous with some having no or strong aromatase activity or progestagen resistance (51). Since the implantation theory moreover cannot explain many other observations of endometriosis, the genetic-epigenetic (GE) theory (55) postulates that the start of a new clone of endometriosis is triggered by a set of GE incidents. Women are born with specific GE characteristics, explaining the hereditary aspect, the susceptibility for developing endometriosis and many associated factors as infertility, endometrial and plasma changes. During life, additional GE incidents can occur during cell division, especially in the endometrium which is the fastest growing tissue. Mistakes are favoured by mutagenic agents like radiation, dioxin pollution and oxidative stress from retrograde menstruation and the upper genital tract and pelvic microbiome. The onset of endometriosis has similarities with the onset of cancer as illustrated by the frequent cancer driver mutation in endometriosis lesions (56). Because of the redundancy of molecular biological pathways, endometriosis only starts when the cumulative set of inherited and acquired GE incidents exceeds a threshold. This explains that each lesion has a different set of GE incidents and that lesions or clones are heterogeneous. Endometriosis thus is not one disease but a myriad of many diseases with typical, cystic and deep endometriosis as the main clinical presentations.

### Pathophysiology: The Growth of Endometriosis

To understand endometriosis, initiation and growth need to be separated (57). A cumulative set of GE incidents not only initiates endometriosis but also determines the subsequent growth in the environment of the peritoneal cavity.

**Figure 2:**
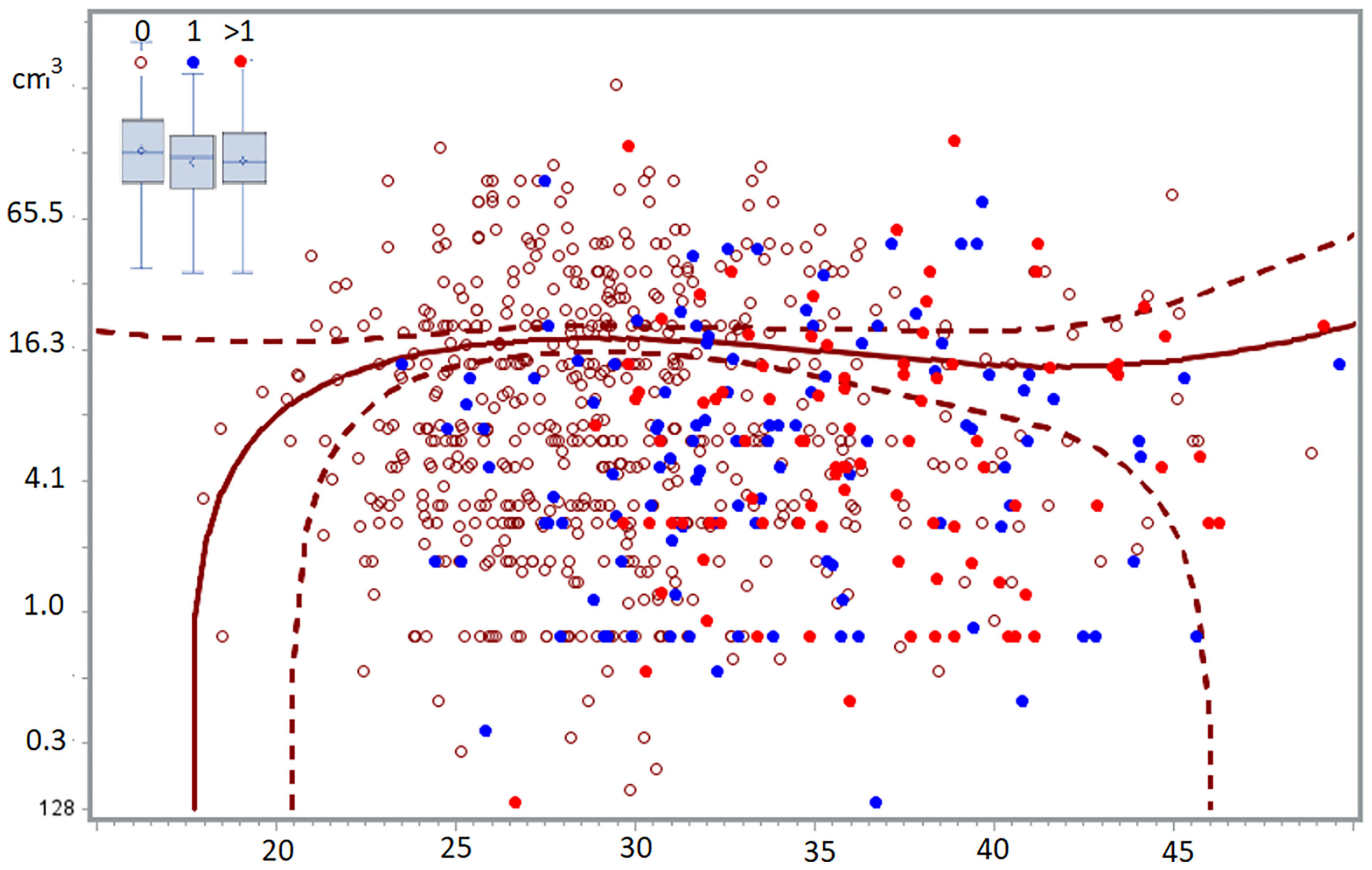
The volume of deep endometriosis lesions (cm^3^) does not vary between 24 and 50 years, for women without, with one or more than 1 child. The least-square regression analysis with 95% confidence limits indicate women without children. In the insert, the boxplots of women with 0, 1 or >1 children demonstrated the absence of a difference between the 3 groups (58).

Most subtle lesions disappear spontaneously and can be considered normal endometrium in an abnormal location. The natural history of typical, cystic and deep endometriosis lesions seems self-limiting since the variability of their severity does not increase with age (**Figure 2**) (58). After a period of growth, lesions stop growing and become inactive, probably because of fibrosis, because of the immunologic reaction and the repetitive trauma and repair of bleeding. This is comparable with a war of trenches between the body and the endometriosis lesions (**Figure 1**), similar to an abscess: the army cannot get in but the enemy cannot get out. Important is that the fibrosis thus belongs to the body, as supported by an endometriosis infiltration of only 1-2 mm in the wall of cystic ovarian endometriosis (59).

That subtle lesions are mostly active and proliferative, notwithstanding the high progesterone concentrations in peritoneal fluid, suggest progesterone resistance (60).

### Endometriosis Associated Events: Cause or Consequence

Endometriosis is associated with infertility, pain, changes in immunology, endometrium, peritoneal fluid and plasma and with pregnancy complications. These can be viewed as the cause or as the consequence of endometriosis or as having a common origin. Although data of changes after surgery could be important for understanding, they, unfortunately, are scarce. CA125 is increased in endometriosis and decreases after surgery suggesting it is a consequence of endometriosis. The decreased natural killer cell activity remains decreased after surgery suggesting it might be a co-factor in initiating endometriosis since present previously (61). Also, pregnancy-associated complications remain unchanged after surgery (62).

### Puberty and the Natural History of Endometriosis

The endocrine and the peritoneal environment change abruptly at puberty and the peritoneal microbiome changes after initiating sexual activity. It is therefore logical, as supported by data, that susceptible women will have the highest risk of developing endometriosis early in life, and that the risk will decrease progressively in the remaining group. After age 30, the risk of initiating endometriosis becomes small (58).

### Why Does Endometriosis Cause Pain and Neuroinflammation at a Distance?

Inflammation can explain endometriosis-associated pain, since half of the subtle and typical, most of the cystic ovarian and almost all of the deep lesions cause some, severe and very severe pain respectively (49). Clinically, 70% of the women with subtle or typical lesions and some 5% with deep lesions do not cause pain.

**Figure 3:**
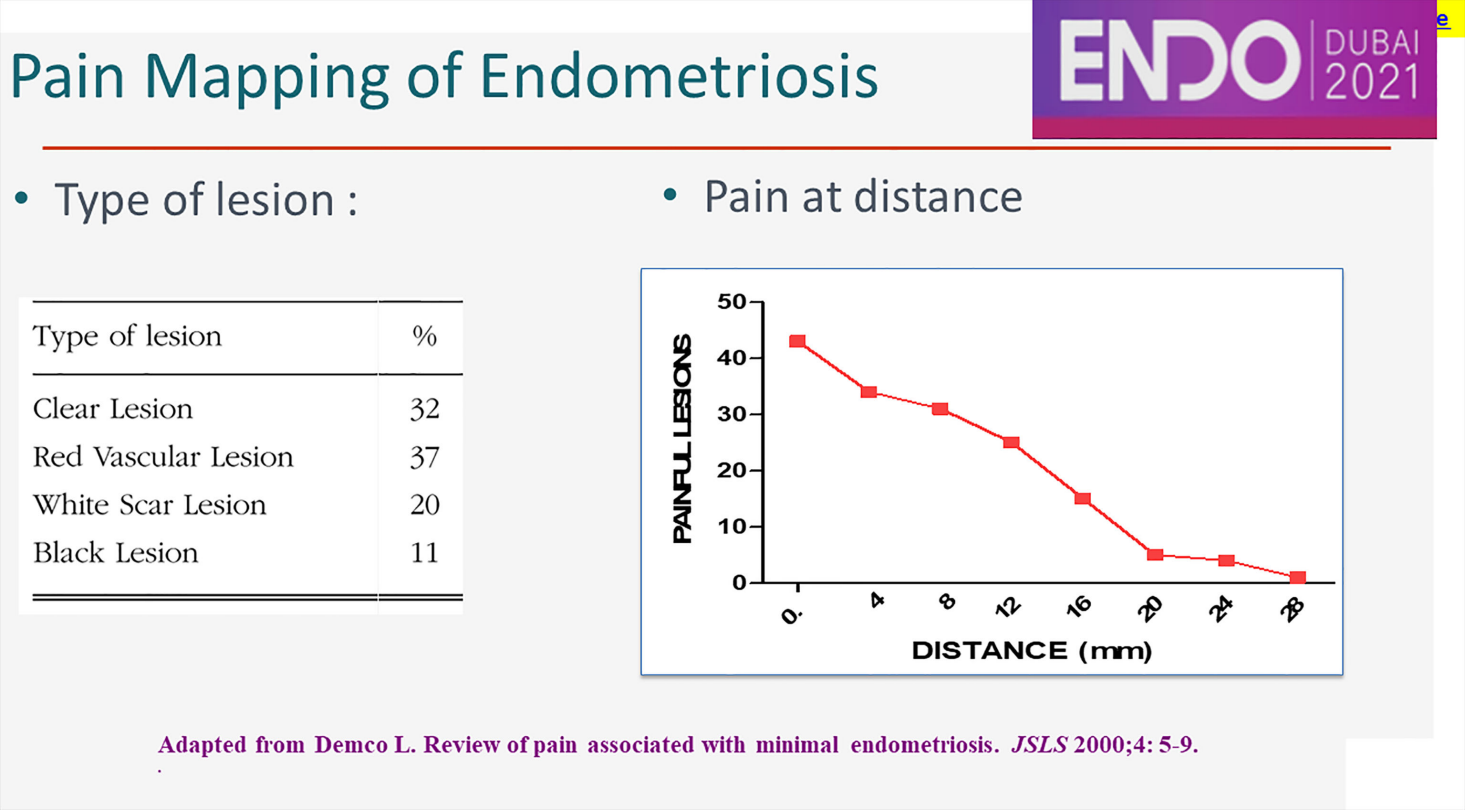
Pain at distance.

The peritoneum around peritoneal lesions is painful during conscious pain mapping up to a distance of 28 mm (**Figure 3**). The mechanism of this neuroinflammation at distance is not known. The clinical importance is that most somatic and sympathetic nerves in the pelvis are within 2.8 mm from the peritoneal cavity (63). This explains that cyclic sciatalgia generally disappears after excision of peritoneal pockets (64), the bottom being close to the obturator nerve (63) and the sciatic nerve.

## Diagnosis of Endometriosis

### Severity and Classification of Endometriosis

The severity of superficial, cystic ovarian and deep endometriosis is highly variable as evaluated by pelvic area, the diameter of cysts, and the volume and localisation of deep endometriosis. However, classifications do not reflect this variability.

The most widely used rASRM classification (50) was proposed in 1968, as an update of the Acosta (65) and Kistner (66) classifications. They are based on the Sampson implantation theory assuming one disease with progression from minor to more severe lesions, and subtle lesions and deep endometriosis were not yet recognised. Also, subsequent updates failed to include them. An analysis of the ASRM classification (67) demonstrated that in over 95% of women, AFS classes I and II were superficial lesions with a total area of less or more than 3cm in diameter and that AFS III and IV were cystic ovarian endometriosis either unilateral and small or bilateral and big. Adhesions and cystic ovarian endometriosis are so strongly associated that adhesions do not need to be be scored separately (67). Since deep endometriosis are mainly found in AFS II it was suggested to classify them separately (68). Since endometriosis has become considered as 3 or 4 different diseases, it is logical to score the severity of each of them separately, as done in the new #ENZIAN score (69).

Unfortunately, no classification has been validated by demonstrating a clinically useful predictive value. All classifications being based on surgical and clinical judgement, it is not surprising to find repetitively the suggestion to distinguish pelvic areas of less or more than 3cm in diameter, cystic ovarian endometriosis of 3, 3 to 6-7, and more than 6-7 cm in diameter and deep endometriosis lesions of less of more than 3cm in diameter. However to define the severity of deep endometriosis severity both the volume, and the area is used. We prefer to define severity by the volume as evaluated at the end of excision when most lesions are spherical by retraction. Imaging oriented surgeons often describe severity by 2 dimensions and define deep endometriosis as deeper than 5mm (40). To highlight the difference, a nodule of 4*4*4 cm is a nodule of 33 cm^3^; for a plaque of 4*4 cm, called a nodule since infiltrating more than 5mm in one point, 8 cm^3^ would be a gross overestimation of the volume.

### Suspicion and Diagnosis of Endometriosis

Endometriosis is suspected because of the type and severity of the symptoms. A clinical exam detects only half of larger deep lesions (70). Imaging is the method of choice for diagnosing cystic ovarian endometriosis and suspecting deep endometriosis. However, neither clinical exam nor imaging can exclude smaller and superficial lesions. Therefore the indication to perform a laparoscopy because of suspected endometriosis remains a clinical decision, knowing that a woman with infertility or chronic pain has a 50% probability of having typical endometriosis and over 70% of having subtle lesions.

Important clinical elements are the knowledge that ovarian pain is felt in the right or left hypogastric area with frequent radiation to the back and the anterior part of the thigh up to the knee, that perineal pain is pathognomonic for bowel involvement (personal communication, P.koninckx), and that isolated back-pain is not gynaecological.

**Figure 4:**
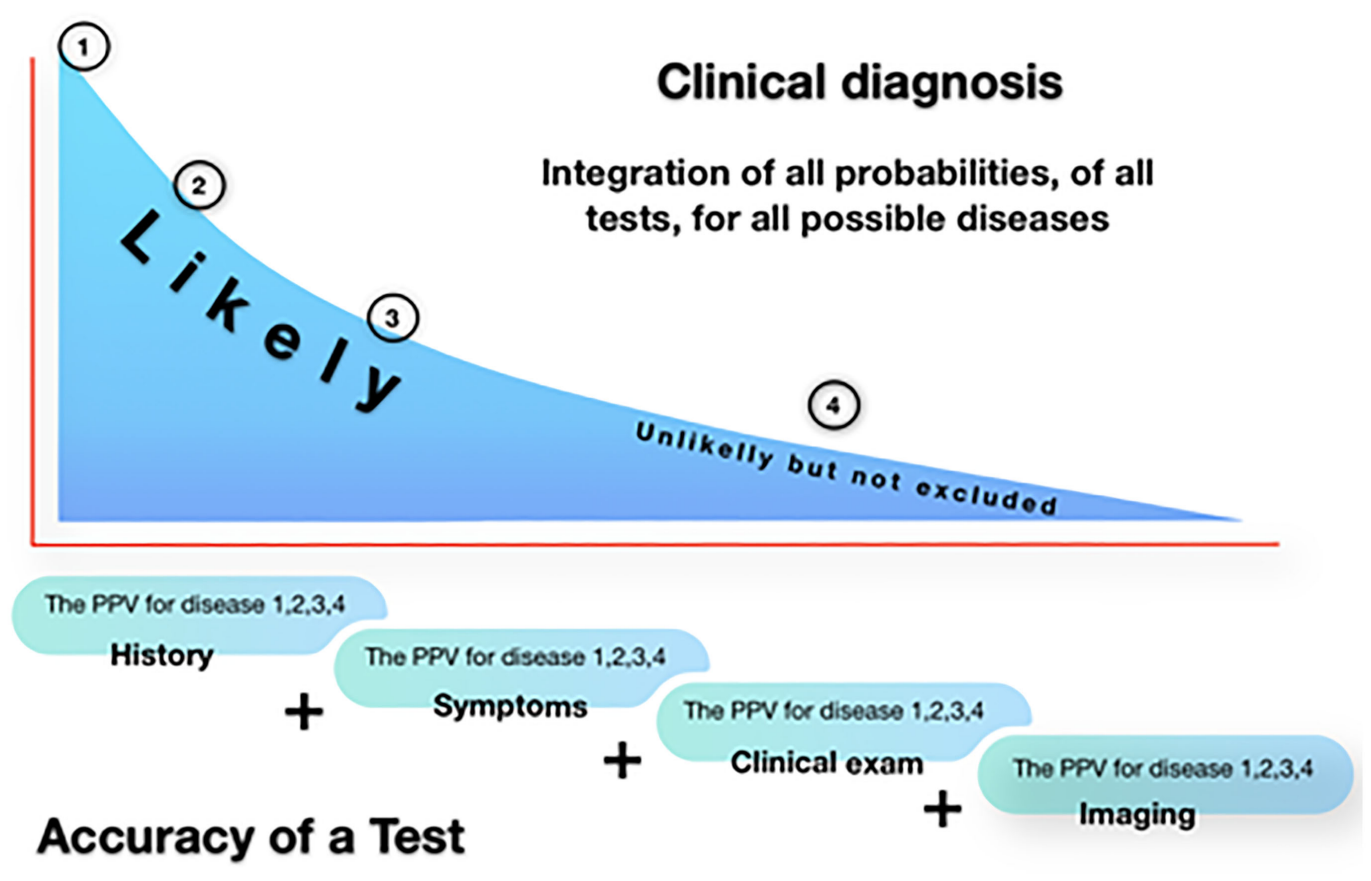
The Clincal diagnosis is an art. History, symptoms, clinical exam and imaging are combined by the clinician into a series of probabilities for the many potential diagnoses. This integration aided by experience could theoretically be replaced by the calculation of the combined predictive values, with their margins of error and confidence limits, of each test for each potential diagnosis.

The value of imaging is not clear. Imaging with ultrasound or MRI is the method of choice to detect cystic ovarian endometriosis. However, imaging cannot distinguish reliably between endometriosis and ovarian cancer (71), which is a clinical problem in older women, especially after menopause. For the diagnosis of deep endometriosis (72) and the discrepancy between the clinical belief in imaging and the data the value of a test needs to be understood (**Figure 4**). The precision and confidence limits of imaging are rarely considered, although a 95% sensitivity obtained in 100 women has confidence limits of 85% to 97%. The predictive value of any diagnostic test decreases when prevalence is low. Simple calculations demonstrate that a test with 99% sensitivity and specificity results in 50% false positives for a disease occurring in 1%. In 100 women with the disease, 99 will be diagnosed, but a 1% mistake in 10.000 women without the disease generates 100 false positives (72). Unfortunately, the prevalence of deep endometriosis is rarely taken into account and, sensitivities and specificities have not been stratified by dimension and localisation of the lesions. Therefore, the predictive values and confidence limits of imaging for the diagnosis of deep endometriosis are limited, even with accuracies of 95 to 99% for all types of lesions taken together. Our interpretation of this apparent contradiction between clinical belief and rather low predictive values, is that decision making is based on an experience-based mix of symptoms and image information, with so-called soft signs. This experience is comparable to the clinical, artificial intelligence like integration of imaging and history and symptoms and clinical exam of the patient. Therefore medicine remains an art, translating the predictive values of age, symptoms, exams, imaging, the history and the eventual other co-morbidities into a decision.

It is a clinical decision based on all available information, with pain severity, radiation and characteristics and imaging being one of them, to decide to do a diagnostic laparoscopy, to initiate medical therapy or to repeat the exams.

### Common Mistakes and Problems of Diagnosis

A common mistake is that endometriosis can be excluded because of negative exams including imaging resulting in the well-known diagnostic delay of diagnosis in endometriosis. Another problem is that diagnostic laparoscopy is avoided and postponed, especially in younger women, since the severity of endometriosis is difficult to predict and surgery can be unexpectedly difficult. As a result, medical therapy is often given for many years without a diagnosis, based on the never demonstrated belief that medical therapy prevents the progression of endometriosis. A third problem is a widely held belief that endometriosis is a recurrent disease and that early surgery risks requiring repeat surgery later with more adhesions and fertility damage.

## Surgery for Endometriosisand Pelvic Pain

### Preparation for Surgery

After the clinical decision to perform a diagnostic laparoscopy, the second decision is whether surgery will be performed during this intervention or postponed for a second intervention, eventually after referral. When surgery is anticipated, it is important to estimate the surgical difficulty and risks, and the anticipated type of surgery. These are needed to inform the patient, obtain informed consent and plan the intervention.

A major problem is an apparent discrepancy between positive and negative predictive values of symptoms, exams and imaging, in comparison with their clinical interpretation based on experience as discussed previously (**Figure 4**). A full description of all aspects being beyond the scope of this article, only the ureter and bowel invasion and stenosis will be discussed. Hydronephrosis, needing a preoperative double J stent (73, 74), can be excluded by transvaginal ultrasound of the ureter and ultrasound of the kidney, an IVP no longer being required. To predict the degree of bowel stenosis, we still ask for a contrast enema. However, many experienced clinicians consider the preoperative ultrasound or MRI evaluation sufficient to plan surgery, eventually at the cost of a slightly higher incidence of bowel resections. Similar arguments can be made concerning the importance of the depth of infiltration of endometriosis in the muscularis of the rectum, of the location of the deep endometriosis nodule and the mobility of the genital organs and bowels and adherences. This illustrates the discrepancy between the predictive value of exams taking into account confidence limits and prevalences and the clinical decision based on experience and intuition using all elements as symptoms, exams, age, history and other morbidities.

Evidence-based medicine is a similar discussion. We consider the value of the consensus opinion of experts, high-ranking in the pyramid of evidence used (75). This consensus opinion grows over many years during meetings, discussions, congresses and (video) conference calls and emails, between friends or colleagues. A lengthy discussion on EBM and the diagnostic accuracy, predictive values and the surgical difficulty could be summarised as follows. If a deep endometriosis nodule of more than 3*3*3 cm, or a volume of more than 14 cm3, is anticipated the intervention risks to be lengthy and difficult. Lesions of less than 2*2*2 cm are rarely difficult except when located in the sigmoid.

A coloscopy rarely adds to the diagnosis and is therefore not very useful even in women with monthly rectal bleeding. The use of preoperative antibiotics is unclear but seems supported by recent evidence of endometriosis and increased infection risks (30). The use of preoperative medical treatment of endometriosis to reduce the volume of endometriosis, although being discussed for many years, has no proven benefit and could increase the risk of missing endometriosis lesions.

### Surgery of Endometriosis

Subtle endometriosis needs to be destroyed since some lesions are painful and some might progress to more severe disease. Since these lesions are small and superficial, CO2 laser vaporisation or bipolar coagulation are the methods of choice.

Typical lesions can be vaporized with a CO2 laser or need to be excised since the variable depth of invasion makes bipolar coagulation less reliable. The efficacy of excising large areas of normal looking peritoneum has never been proven.

Surgery of cystic ovarian endometriosis varies with the diameter, number and localisation of the cysts and the associated adhesions. The following principles need to be balanced by surgical judgement. Small cystic ovarian endometrioma’s in young women can be treated by transculdo-hydro-laparoscopy (THL) (76, 77), having the advantage of being minimally adhesiogenic. In women with infertility, surgery of endometrioma’s less than 3cm does not improve the success rate of IVF. Excision of cystic ovarian endometriosis has a lower recurrence rate of 5% than superficial destruction with recurrence rates around 20% (78, 79). However, excision can be associated with more ovarian damage if the surgeon is less experienced, ie the singer or the song (80). For endometrioma’s larger than 6 cm situated within the ovary, excision leaves a thin rim of ovarian tissue with poor vascularisation. Therefore, either an ovariectomy is performed, or in women with infertility, surgery needs to be done during a second intervention after reduction of the volume by a marsupialisation and 3 months of GnRH therapy. In these large cysts, complete superficial destruction is difficult and irrealistic considering that the surface of a 6 cm endometrioma is 113 cm^2^.

For deep endometriosis, surgical excision is the method of choice. As described in recent reviews (81, 82), for rectum lesions a conservative excision without a bowel resection is almost always feasible but at the cost of eventually a lengthy muscularis suture. A less complete excision, finished by a wedge resection using a circular stapler, seems the way forward (83, 84) as suggested by clinical observation. Bowel resection and anastomosis are indicated for very large nodules of more than 4*4*4cm and with a bowel occlusion of more than 50% over more than 2cm. Large bowel resections, based on vascularisation have the advantage of being a well-known technique (85) but low rectum resections are associated with 30% lifelong bowel, bladder, and probably sexual complications. Recently a minimal and short bowel resection seems to be preferable (A. Wattiez, personal communication). Since side effects of a sigmoid resection are minimal, and excision is technically difficult, liberal use of sigmoid resection is advocated.

It remains debated whether excision should be done close to the endometriosis or with some safety margins. For deep endometriosis of the vaginal cuff or the bladder, we emphasise the completeness of excision, since both tissues heal well, and since in our experience, almost all recurrences occurred in the vaginal cuff. When excising a nodule from the bowel or the ureter we prefer to leave a rim of fibrosis since recurrence rates are similar after excision, short bowel resection and large bowel resections. This argument is equally valid for excisions around neural structures.

Nerve-sparing is a well-established technique in oncologic surgery which aims at a broad resection of all potentially invaded tissues around cancer. Endometriosis surgery is based on nerve anatomy and avoiding damage. When endometriosis infiltrates a nerve it is a surgical decision to balance completeness of surgery with nerve damage.

A difficult discussion is pain from, and exploration or surgery of large somatic nerves, and by extension of sympathetic nerves. Some endometriosis lesions do not cause pain, but some can cause neuroinflammation and pain at a distance up to 28 mm (49). Unfortunately, we cannot yet evaluate which lesions cause neuroinflammation at distance. Giving the risks of nerve exploration, we consider that this should be done with restraint (63).

### Prevention of Complications and Adhesions

Endometriosis surgery needs to balance completeness of excision and prevention of complications during and after surgery while preserving bowel and bladder function and fertility. This multifactorial decision needs surgical judgment, based on the knowledge of anatomy and of the disease endometriosis, and varies with the skills and experience of the surgeon.

At the end of the surgery, we run a checklist. If the bowel was opened it seems wise to give antibiotics for a week and to leave a drain for 3 to 5 days. If the vagina was opened a one-shot of antibiotics is given. Considering the increased risk of postoperative infection in women with endometriosis (30) antibiotic prophylaxis at the beginning of surgery might be considered. After a ureter lesion or ureter surgery, a double J stent is left for 6 weeks. An omental flap close to a lesion could enhance healing, although not demonstrated. After bladder surgery, a large bladder catheter permitting to evacuate clots is left for 10 to 20 days. A daily CRP is advocated to ascertain a decrease from day 3 after surgery onwards and clinically the patient should always improve; otherwise, an early second-look repeat laparoscopy should be considered because of blood, infection or a late bowel leak (86).

Prevention of postoperative adhesions (36, 87, 88) is important because of the associated infertility and the long term complications of bowel obstruction and pain. Besides increasing with the duration of surgery and the surgical trauma, adhesions increase with infection and blood in the peritoneal cavity. Unfortunately, because adherent to the microvilli of the mesothelial cells bacteriae cannot completely be removed by washing. The prevention of adhesions was described for microsurgery. Recently the underlying mechanism of acute inflammation was understood and described comprehensively as peritoneal conditioning. Duration of surgery and insufflation pressure are important since CO2 causes a duration and insufflation pressure-dependent acute inflammation in the entire abdomen, for which 10% of N2O should be added to the CO2 pneumoperitoneum. Gentle tissue handling minimises grasping and manipulation, and the amount of dead tissue left behind should be reduced. The latter points to knowing how to use energy, and to balance the extent of coagulation with the prevention of bleeding. Desiccation needs to be prevented and the temperature in the abdominal cavity should be kept below 31°C. At the end of the surgery, the beneficial effect of 5 mg of dexamethasone needs to be balanced with the risk of infection. Similarly, the use of an adhesion barrier and the risk of infection has to be considered. Although each aspect of peritoneal conditioning is well established and since full conditioning permits adhesion free surgery, the implementation in the individual patient is a surgical judgment equilibrating opposing effects as insufflation pressure and working space, duration of surgery and type of intervention, extensive coagulation and leaving devascularised tissue and adhesion barriers and the risk of infection. This equilibrium may vary for the individual patient and with the skills of the surgeon.

## Medical Therapy of Endometriosis

Endometrium and endometriosis need estrogens to grow, which explains the efficacy of reducing circulating estrogens with GNRH. However, some endometriosis lesions do not need circulating estrogens (89) because of the aromatase activity in the lesion. Since progesterone stops endometrial growth and induces secretory changes, progestins and oestro-progestins are widely used as a therapy for pain associated with endometriosis. However, some lesions have a strong progesterone resistance (51). This individual variability of these clonal lesions, with variable aromatase activity and progesterone resistance, has not yet been integrated into the medical treatment of endometriosis. Moreover, the evaluation of the efficacy of therapy can no longer be evaluated by traditional statistics as means and standard deviations since they require a homogeneous group, and since they do not permit the detection of subgroups with different behaviour.

Medical therapy does not improve fertility. Medical therapy inactivates and decreases the volume of some endometriosis, lesions and endometriosis-associated pain is believed to decrease. However, these trials were not and cannot be blinded since treatment is recognised by the woman especially when affecting menstruation. Moreover, dysmenorrhea being absent since in amenorrhoea, global pain scores should not be used (90).

It is widely believed that medical therapy prevents the growth of endometriosis. Our recent observation that endometriosis is a self-limiting disease since the severity of endometriosis lesions does not vary with age or with previous pregnancies challenges this concept (91).

We do not use medical therapy before surgery since the risk of missing lesions is considered more important than the potential benefit of a decreased vascularisation and a smaller volume. When spastic dysmenorrhoea is suspected, medical therapy can be given for a short period and if 100% effective continued. Since medical therapy is not effective in all lesions and considering the many causes of pelvic pain besides endometriosis, we avoid giving medical therapy without a diagnosis, i.e. without a laparoscopy. When medical therapy is not 100% effective, a laparoscopy seems indicated.

New is the likely role of oxidative stress in initiating endometriosis, and therefore the prevention of new lesions by reducing retrograde menstruation and pelvic infections by giving oestro-progestins continuously. Although the efficacy of this prevention remains to be demonstrated, it has become our standard practise with eventually some antioxidants during menstruation.

## Conclusions and Discussion

Diagnosis and therapy of endometriosis should be based on the knowledge of the disease and observations, not on beliefs. Our understanding of endometriosis is changing rapidly, and this needs to be integrated with clinical management. This will initially be done by slightly different approaches, which will converge into consensus opinions, and later important aspects will be refined and demonstrated in trials. Unfortunately, RCT’s are not well suited for a variable disease as endometriosis with complex surgical interventions and multimorbidity (92).

This review is an attempt to integrate the new understanding of endometriosis with the consensus opinion of experts on diagnosis and therapy. Aspects, which we consider too speculative today were not integrated but will be discussed briefly.

Endometriosis lesions cause an inflammatory reaction and fibrosis, which can be compared to an abscess or a war of trenches, confining but not eradicating the disease. The fibrosis around the disease belongs to the body. The endometriosis can infiltrate superficially this fibrosis as demonstrated for cystic ovarian endometriosis, with infiltration of the capsule limited to 1 or 2 mm. This concept suggests that fibrosis needs not to be removed as we suggested years ago based on clinical observation, that a ‘rim of fibrosis could be left during surgery’ (93). For cystic ovarian endometriosis, superficial destruction seems logical provided that destruction of the entire surface can be achieved. Alcoholisation is attractive (94) but efficacy is still poorly demonstrated. For deep endometriosis, it suggests excision that follows the border of the lesions.

Endometriosis can cause pain, but not all lesions are painful, and not all painful lesions cause pain at distance. We think that this is caused by variable chemical neuroinflammation with an effect varying with its concentration and decreasing with distance. However, satellite lesions of microscopical endometriosis cannot be excluded. The clinical importance is that the former hypothesis does not require safety margins when excising endometriosis, whereas the latter hypothesis suggests safety margins as used in the past.

The risk of initiating endometriosis lesions varies with the genetic predisposition and with the oxidative stress from retrograde menstruation and the pelvic microbiome. It is attractive to consider that in susceptible women this risk is highest after puberty and after initiating sexual activity (58), i.e. during or early after puberty. In the remaining group, the risk will progressively decrease, becoming low after 30 years of age, as demonstrated recently. Therefore we should focus on susceptibility, and clinically giving combined oestro-progestins to susceptible women early in life, before they develop endometriosis, to decrease retrograde menstruation and ascending infections, might be considered. That the peritoneal microbiome is influenced by the gastrointestinal microbiome opens a series of new treatments of endometriosis by exercise and food intake. This concept is also important for adolescent endometriosis (76, 95). Today, early diagnosis and surgery are avoided because of the risk of recurrences and repeat surgery and more damage. This would change if the prevention of new lesions will be demonstrated to be effective. Especially THL should be considered for women with small cystic ovarian endometriosis (76).

The diagnosis of endometriosis and the prediction of the surgical difficulty are important. However, we should realise that our actual analytic approach, describing the sensitivity and specificity of each diagnostic method is not very helpful. The clinician needs to know the predictive value of a diagnosis or the absence of a diagnosis or alternative diagnoses in the individual patient. This requires the combination of all PPVs and NPVs of symptoms, clinical exams, blood tests and imaging in an individual woman with her specific heredity, antecedents and age, and taking into account precision of estimation of each test and prevalence of the disease. It is obvious that the data to calculate the probability of the diagnosis of each form of endometriosis do not exist and will not be available soon. However, this integration is what the brain of the clinician does when taking decisions after considering the most likely and the rare diagnoses. This explains the apparent discrepancy between the perceived clinical usefulness and the demonstrated accuracy of symptoms and exams and imaging of endometriosis.

Rare clinical presentations as juvenile cystic adenomyoma’s (96) and peritoneal pockets (63) and their treatment (97) are not discussed since beyond the scope of this manuscript.

In conclusion, endometriosis diagnosis and treatment is a clinical art, based upon knowledge, experience and skills.

## Author Contributions

Pathophysiology, diagnosis, and treatment of endometriosis were discussed in detail with all authors. All authors read, commented, and approved the manuscript.

## Funding

Frontiers DSC-07031402521PRD.

## Conflict of Interest

The authors declare that the research was conducted in the absence of any commercial or financial relationships that could be construed as a potential conflict of interest.

## Publisher’s Note

All claims expressed in this article are solely those of the authors and do not necessarily represent those of their affiliated organizations, or those of the publisher, the editors and the reviewers. Any product that may be evaluated in this article, or claim that may be made by its manufacturer, is not guaranteed or endorsed by the publisher.
